# Structure of Microemulsion Formulated with Monoacylglycerols in the Presence of Polyols and Ethanol

**DOI:** 10.1007/s11743-014-1618-x

**Published:** 2014-08-02

**Authors:** Patrycja Szumała

**Affiliations:** Department of Fats and Detergents Technology, Chemical Faculty, Gdansk University of Technology, Narutowicza 11/12, Gdansk, 80-233 Poland

**Keywords:** Microemulsion, Monoacylglycerol, Polyol, Ethanol, DSC, DLS

## Abstract

The influence of polyols as cosurfactants (propylene glycol PG; glycerol G) and short chain alcohol as a cosolvent (ethanol EtOH) on the formation and solubilization capacity of the systems: hexadecane/monoacylglycerols (MAG)/polyol/water:EtOH, at 60 °C, was investigated. Electrical conductivity measurement, and the DSC method were applied to determine the structure and type of microemulsions formed. The dimension of the droplets was characterized by DLS. It has been stated that concentration of EtOH has a strong influence on the shape and extend the microemulsion areas and helps to avoid rigid structures such as gels, precipitates, and liquid crystals. It was found that, depending on the concentration of five-component systems, it was possible to obtain fully diluted microemulsions with dispersed particles size distribution ranging from 5 to 30 nm. Studied systems are changing the w/o structure into a bicontinuous system. The results of electrical conductivity showed that the electrical percolation threshold is dependent on the hydration of polar head groups in the whole system and the less rigid interfacial film due to the intercalation of ethanol. In addition, the surfactant/alcohol/polyol can strongly bind water in the inner phase so that it freezes below −10 °C and acts in part as ‘bound’ water. In the systems containing more than 50 mass% of polyols, with respect to the water, the all the water was non-freezable. Propylene glycol and glycerol are cryoprotectants protecting biological systems from massive ice crystallization, since they lower the freezing point of water.

## Introduction

Isotropic, spontaneous forming, microstructured liquid phases named microemulsions are composed of water, an oil phase and a surfactant, or more generally a mixture of surfactant and cosurfactant. Since their discovery [1], microemulsions have been the subject of numerous studies as regards their singular physicochemical, rheological and structural properties as well as the ensuing practical applications [2, 3]. Valuable information concerning the physicochemical properties of microemulsions has been provided by various studies such as: thermodynamic properties (phase diagrams, component activity), transport properties (conductivity, diffusion) and structural properties (tracer and NMR self-diffusion studies, light-scattering and dielectric measurements). Nonionic surfactants that are extensively used in microemulsions (ME) production are polyoxyethylene sorbitan fatty acid esters (Tween) [4–6]. However, even ethoxylated surfactant based microemulsions are not as well studied and well known as ionic ones and the mechanism, for example, of structure transition in nonionic microemulsions is still not fully understood. It is very important and necessary to resolve the mechanism of phase behavior, because there are advantages in using nonionic microemulsions in many applications compared to ionic ones, such as in nanoparticle growth applications in which it is beneficial to avoid unfavorable interactions between ionic reactants and surfactant counterions [7]. However, only a limited amount of this kind of work was conducted on the polyol type of nonionic surfactants.

Monoacylglycerols (MAG) are a class of polar food lipids obtained from natural raw materials, namely glycerol and fatty acids and they are widely used in many products of the food industry and cosmetics [8, 9], and has been designated by FDA to be a GRAS (Generally Recognized As Safe) food additive. MAG are able to undergo interfacial crystallization (at oil–water and air–water interfaces) and to form various liquid-crystalline (LC) phases with water, which have an influence on their wide use in many food applications, cosmetic products and pharmaceutical formulations [10–12].

In the past decade, the self-assembly of different surfactants in polar nonaqueous solvents like propylene glycol (PG), glycerol, and formamide has been investigated to explore how these solvents influence the micellization process and the formation of LC phases. These solvents, like water, form hydrogen bonds, have relatively high dielectric constants, and are immiscible with hydrocarbon solvents. When these solvents are used as water substitutes, their penetration into the surfactant interface leads to smaller or no LC phase regions [13–15]. Regarding cosurfactants, short-chain alcohols are the most commonly used cosurfactants in microemulsion formulations; however, ethanol is often referred to as a cosolvent. A short chain cosurfactant/cosolvent helps to avoid rigid structures such as gels, precipitates, and liquid crystals (LC), and to extend the microemulsion area [16, 17].

The recognition of the phase behavior and water solubilization capacity in the model system is expected to play a key role in producing ME as delivery systems, particularly with nonionic surfactants. In our previews study [18], we obtained four component systems based on MAG, that are homogeneous waxy solids at room temperature and, therefore they can immobilize, protect, inactivate, or slow down the activities of solubilized water-soluble matter during storage. The solid system will liquefy and convert or restore spontaneously into ME upon heating. On the findings existing in the scientific literature on the role of ethanol, polyols and surfactants in microemulsions, the present work aims to study in detail different factors affecting the phase behavior to obtain the desired formulations. This paper reports on the phase diagram, percolation threshold and sub-zero temperature behavior of nonionic microemulsions in the presence of PG, glycerol, and ethanol.

## Experimental Procedures

### Materials

Molecular distilled monoacylglycerols (MAG >95 %) (MYVEROL RX GMS 95P, a mixture of glycerol palmitic (57,8 %) stearic (37.3 %) and myristic (1.3 %) acid monoesters, were purchased from KERRY BIO-SCIENCE (Netherlands). Ethyl alcohol (EtOH; 96 % pure) and propylene glycol (PG; 99.5 % purity) were obtained from POCH (Poland). Glycerol (G; waterless 96 % purity) was obtained from EUROCHEM BGD (Poland). Hexadecane (purity >98 %) was obtained from FLUKA (Poland). The water used for the preparation of the systems was purified in a ROpureST/NANOpure system (Barnstead, USA).

### Methods

#### Microemulsion Formation

Microemulsions were prepared in a glass thermostated emulsor using the mechanical stirrer (Heidolph RZR 2021, Germany). The water phase was added dropwise to a mixture of the other components (oil phase, surfactant and polyol) until its solubilization limit was reached, (the system became turbid) under continuous stirring (300 rpm). The temperature of microemulsion preparation was 60 ± 1 °C; chosen with respect to the melting temperature of the compounds, mainly MAG.

Since the behavior of four components is to be depicted on a pseudo-ternary phase diagram, the relative concentration of two of the components must be kept constant for all the experiments. The optimal surfactant-to-cosurfactant mass ratio is found to be 1:1 (MAG:PG) to reduce the concentration of the surfactants in the systems with PG, which is quite common and a constant weight ratio of oil-to-cosurfactant (C_16_H_34_/G was 1/1) in the systems with glycerol.

#### Conductivity Measurement

During the emulsification, the electrical conductivity (conductivity cell used was Tetra Con^®^ 325, WTW, Germany) of the studied systems was continuously measured to determine the type of microemulsion formed. In the case of microemulsions stabilized by nonionic surfactants, a small amount of an aqueous electrolyte must be added for electrical conductivity [14, 15]. Thus, a 0.05 % sodium chloride aqueous solution was used in the preparation of the microemulsion samples in place of a pure water phase. Based on the data obtained, pseudoternary phase diagrams were formulated. The isotropic region was identified when clear and transparent systems were obtained by visual examination of samples. The amounts of water of the corresponding phases behavior in the systems were determined from three preparations and showed a maximum standard deviation of ±1 %.

#### Calorimetric Measurements (DSC)

Thermal analyses were conducted on a Mettler TA3000 calorimeter (Mettler Instrument, Switzerland) equipped with a TC 10 TA processor and a differential scanning calorimeter (DSC) with a model 30 temperature cell. The DSC measurements were carried out as follows: microemulsion samples (15–30 mg) were weighted into 40-μL aluminum pans and immediately hermetically sealed. The samples were rapidly cooled by liquid nitrogen from ambient to −90 °C and then heated at a constant scanning rate (usually 5 °C/min) to 70 °C. An empty pan was used as the reference.

#### Droplet Size

The mean droplet size and size distribution of the microemulsions were determined using a non-invasive back scatter method (NIBS). A Zetasizer Nano ZS (Malvern Instruments, Malvern, United Kingdom) equipped with a helium–neon laser diffraction operating at 633 nm was employed. Measurements were made at given temperatures of the microemulsions formation (60 °C).

## Results and Discussion

### Influence of Ethanol on the Phase Behavior of Microemulsion Based on Monoacylglycerols and Propylene glycol (MAG:PG)

**Fig. 1:**
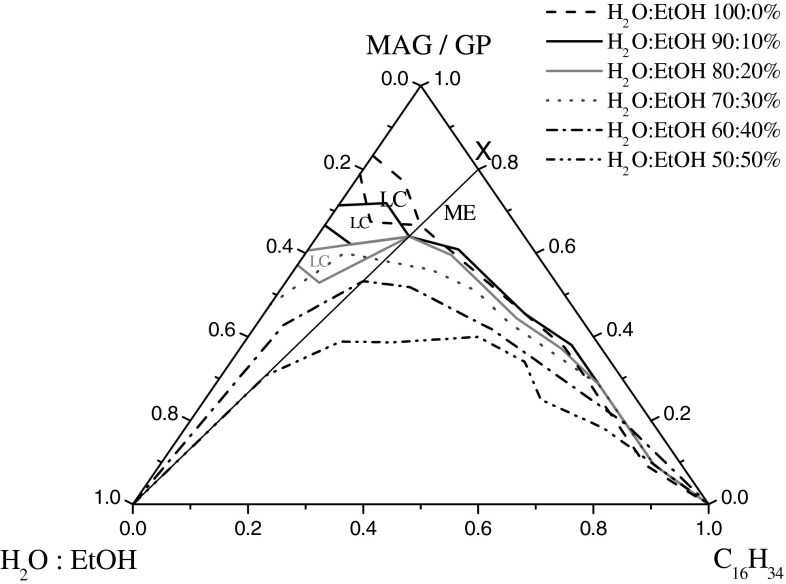
Phase diagram of the hexadecane/monoacylglycerols/propylene glycol/water:EtOH systems. *ME* area of microemulsion; *LC* liquid crystals phase; *X* dilution line

The phase diagram of a five-component system of hexadecane/water/ethanol/MAG/PG is presented in Fig. 1. The polyol:monoacylglycerol weight ratio was held constant at 1:1. The H_2_O:EtOH weight ratios were 90:10 to 50:50 (%). In addition, the system without ethanol is shown. One can clearly see that ME are formed along with LC areas in the systems without and with max. 20 % of EtOH in the water phase. The change of the phase behavior in the presence of different concentration of ethanol is shown. The liquid crystalline phase area decreased as more ethanol was added. The change in the solubilization parameters was dramatic. Increasing the EtOH content (by changing the water/EtOH ratio) resulting in a larger isotropic phase dominating most of the phase diagram area. Moreover, microemulsion with 40 and 50 % ethanol solution can be diluted (X dilution lines “reach” the water corner of the phase diagram). The great advantage of fully or highly water dilutable ME is the fact that they allow a very good dispersion of active substances in a huge volume of water. The studied microemulsions in this article were not diluted with pure water, but with water and ethanol. However, the results obtained provide valuable information, which will be used to develop a bicontinuous structure microemulsion that contains significant amounts of oil and aqueous phase (highly water dilutable ME) with minimal cosurfactants content (in a forthcoming article).

**Fig. 2:**
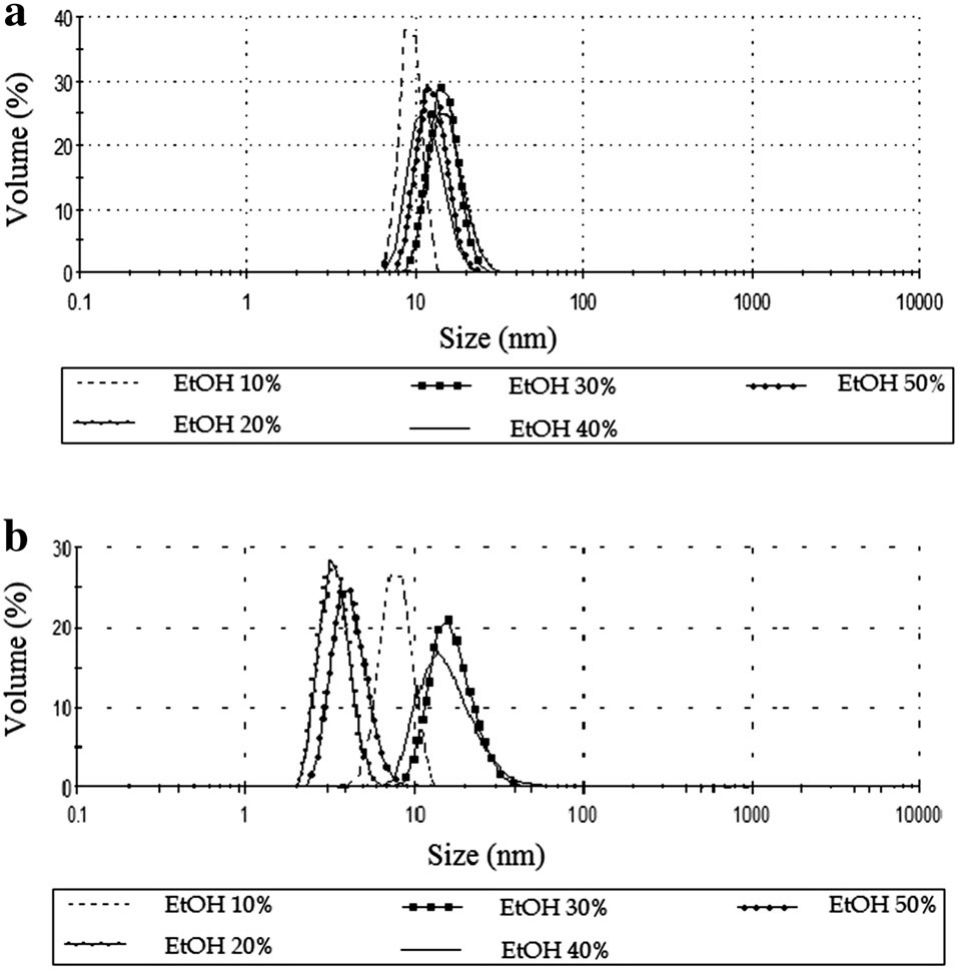
Diameters of the microemulsions droplet obtained by DLS method for the systems with maximum amount of water and with different EtOH concentrations; **a** C_16_H_34_/MAG/PG/water:EtOH; **b** C_16_H_34_:G/MAG/water:EtOH

The change in phase behavior caused by the addition of ethanol and PG can be interpreted in terms of film properties; that is, the flexibility of the surfactant film is increased because the LC phase is destabilized in favor of a microemulsion phase. In addition, the increase in interfacial fluidity for the formation of isotropic microemulsions from organized structure (LC) is clearly an important factor. It is well known that polyols such as PG and short-chain alcohols have a “salting-in” effect and act as cosurfactants [14, 19]. Therefore, it is assumed that a considerable part of PG is incorporated into the surfactant layer and thus will increase the interfacial fluidity, and the other part of PG will decrease the polarity of the water because PG is mainly soluble in water. However, the addition of EtOH, a short chain alcohol and hence very flexible, makes the interfacial film itself more flexible and increases the single phase area. This allows the water droplets to merge and to incorporate more water. When the concentration of ethanol was increased in those systems it was also possible to use smaller amount of surfactant to obtain ME with improved solubilization capacity of water. This is very important in terms of application and economy. It was also stated that ethanol affects the droplet size slightly in the studied systems. Figure 2a shows that in microemulsions stabilized by MAG and PG, particle size distributions were from about 5 to 30 nm.

Another important observation (not shown) was that all of the systems with EtOH solutions from 30 to 50 % are temperature-resistant over a temperature range 40–60 °C. There is no change in the monophasic area. The observed temperature independence of these systems can be a consequence of the nonionic surfactant solubility in the ethanol. Monoacylglycerols are hydrophobic insolubles in water emulsifiers. But they dissolve in ethyl alcohol, propylene glycol and oil phases. In the studied system, at all temperatures, the surfactant is additionally soluble in the aqueous phase (EtOH/water mixture), not just in oil. This effect is important in many detergent or cosmetic products that should remain stable under fluctuating temperatures.

### Influence of Ethanol on the Phase Behavior of Microemulsion Based on Monoacylglycerols and Glycerol

**Fig. 3:**
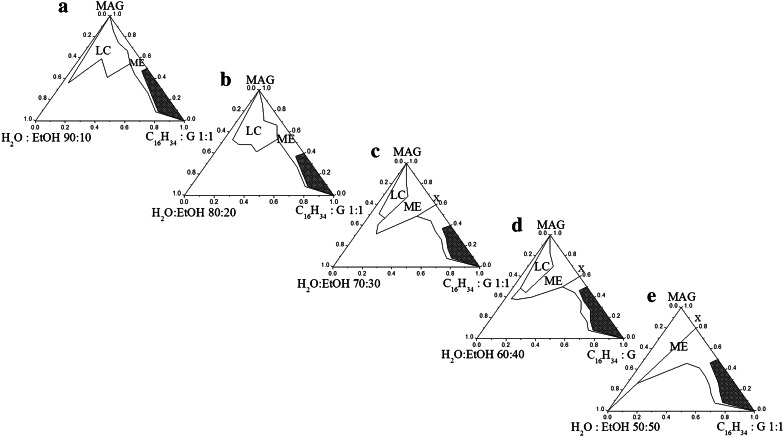
Phase diagrams of the hexadecane/glycerol/monoacylglycerols/water:EtOH systems; **a**–**e** systems which ethanol concentration were 10, 20, 30, 40, 50 % respectively, *ME* area of microemulsion; *LC* liquid crystals phase; *X* dilution line

The behaviour of these five-component systems are described in pseudo-ternary phase diagrams (Fig. 3). The phase diagrams were constructed in the following way. Mixtures of oil, glycerol and surfactant were made. The polyol:oil weight ratio was held constant at 1:1. Mixtures were kept at 60 °C in a water bath. Microemulsion areas were determined in phase diagrams by titrating the oil–glycerol–surfactant phase with the aqueous phase (H_2_O:EtOH weight ratios 90:10 to 50:50). The procedure was changed (constant ratio of oil to polyol instead of surfactant to polyol), because such a composition results in an increase in the aqueous phase systems (when we applied a solid weight ratio of G:MAG, the systems were characterized by negligible amounts of the aqueous phase in ME). Glycerol is known to be a good cosolvent but has no surface activity, and it is not incorporated into the surfactant film [14, 19]. Therefore, the creation of this type of ME requires much larger amounts of surfactant.

Microemulsions based on G show behavior different to that of those made with PG. ME regions are smaller because the LC area is much bigger and turbid systems were observed (the shaded area). In the systems with more than 1:1 surfactant-to-oil+glycerol ratios, the turbid regions were observed after we had added initial amounts of the water phase. The reason for this might be the change in polarity of phases generated due to a large amount of glycerol, thus there is a change in the solubility of the surfactant. After adding a certain volume of the water phase, the transparent systems were observed.

However, the effect of ethanol is similar. In the presence of a small percentage of ethanol, EtOH ≤20, the single phase is very limited and large zones of LC appear. The presence of LC can be attributed to a greater hydration of the interfacial film [16, 17]. This prevents the penetration of the surfactant headgroup into the oil phase. Another aspect is the insufficient amount of ethanol to separate the monoacylglycerol molecules to make the interfacial film more flexible and it prevents the micelles to merge. The solution consists of discrete water droplets within a continuous oil phase. As the alcohol content increases from an water/ethanol weight ratio from 70:30 to 50:50, larger isotropic areas are formed. In addition, the ME area is formed in place of the previously created LC until complete elimination of the liquid crystal structure in a system comprising 50 % ethanol solution. Moreover, microemulsion with 50 % of ethanol can be diluted (line “reach” off the water corner of the phase diagram).

Table 1 shows the solubilization parameters of the system C_16_H_34_:G/MAG/H_2_O:EtOH as a function of the ethanol concentration at different hexadecane:glycerol/monoacylglycerols ratios. The amounts of water increase considerably with increasing ethanol concentration in the systems. The results indicated that the ME water solubilizing abilities based on G were greater than that of the PG. However, to obtain this ME I had to introduce significantly more surfactant (compared to the ME based on PG).

**Table 1:** Solubilization parameters of the systems based: C_16_H_34_:G/MAG/H_2_O:EtOH

H_2_O:EtOH (weight ratio)	C_16_H_34_:G/MAG (weight ratio)	Oil (%)	Sm (%)	Wm (%)	Wm/Sm
90:10	2/3	3.3	54.5	8.2	0.15
80:20	2/3	4.8	53.3	8.9	0.17
70:30	3/7	6.8	31.8	38.7	1.22
60:40	1/4	17.7	38.1	31.4	0.82
50:50	3/7	18.2	26.7	33.3	1.25
50:50	1/9			Fully diluted	

*Wm* maximum amount of solubilized water, *Sm* amount of surfactants needed to obtain maximum solubilization

It was also stated that ethanol affects the droplet size in the studied systems. Figure 2b shows that in microemulsions with glycerol in addition of 20 and 50 % EtOH, particle size distributions were from about 5–10 nm. When the system contained 30 and 40 % ethanol, diameters of the microemulsions droplet were from 10 to 30 nm. As before, the droplet size does not exceed 30 nm, however, the various systems, depending on the ethanol content, have more varied particle sizes, compared to systems with propylene glycol.

### Thermodynamics of Water Solubilization

Microemulsions were formulated upon water solubilization in mixed hexadecane/surfactant:PG and hexadecane:glycerol/surfactant medium. The corresponding free energy of dissolution (Δ*G*_s_^o^) at a constant temperature can be obtained from the relation [20–22]:

$$\Delta G_{\text{s}}^{\text{o}} { = - }RT{ \ln }X_{\text{d}}$$

where *X*_d_ is the mole fraction of the dispersed phase (in our case water) and *R* is the gas constant (m^3^ Pa/kg K). The estimation of (Δ*G*_s_^o^ )reported herein was performed based on mole fraction of dispersed water. The free energy of solubilization (Δ*G*_s_^o^) for water-in-oil microemulsions studied were calculated and the values are given in Table 2. It was found that the Δ*G*_s_^o^ values decrease with water content in the water-in-oil microemulsions indicating that adding water to the water-in-oil microemulsions disrupts their organization (shown only for the systems with 10 % of EtOH). We also calculated Δ*G*_s_^o^ values of system with maximum solubilization of water phase and in the presence of 10–50 % ethanol solution. As can be seen, the values of free energy of dissolution are higher for the systems with glycerol, however *X*_d_ are smaller.

**Table 2:** Δ*G*_s_^o^ values of the selected systems for different polyol and solution of ethanol

H_2_O:EtOH (weight ratio)	C_16_H_34_/MAG:PG 2/8 (w/w)		C_16_H_34_:G/MAG 3/7 (w/w)	
	*X*_d_ water mole fraction	Δ*G*_s_^o^ (kJ/mol)	*X*_d_ water mole fraction	Δ*G*_s_^o^ (kJ/mol)
90:10	0.61	1.35		
	0.56	1.59	0.43	2.31
	0.50	1.94	0.38	2.66
	0.40	2.54	0.24	3.97
	0.25	3.82		

	*X*_dmax_		*X*_dmax_	
80:20	0.57	1.56	0.40	2.55
70:30	0.60	1.42	0.48	2.01
60:40	0.59	1.47	0.41	2.47
50:50	0.58	1.50	0.43	2.35

*X*_dmax_ maximum mole fraction of solubilized water

### Electrical Conductivity

The percolation of conductance (both volume and temperature-induced) in w/o microemulsion is an interesting and fascinating phenomenon that has been intensively studied [16, 23–26]. When the volume fraction of the dispersed phase (water) increases to a certain volume, the conductivity is abruptly increased. This increase remains invariable after reaching a maximum value, which is much higher than that for the microemulsions present before this transition occurs. This phenomenon is known as electric percolation, and the moment at which an abrupt transition occurs is termed the percolation threshold. Among the proposed mechanism of electric percolation, the cluster model is therefore more reasonable and has been widely used in recent years [25–28]. The droplet clusters appear and increase very rapidly above the percolation threshold. The abrupt increase in electric conductivity is attributed to either ‘‘hopping’’ of surfactant ions from droplet to droplet within droplet clusters [28, 29] or the transfer of counterions from one droplet to another through water channels opening between droplets during ‘‘sticky’’ collisions or through transient merging of droplets.

**Fig. 4:**
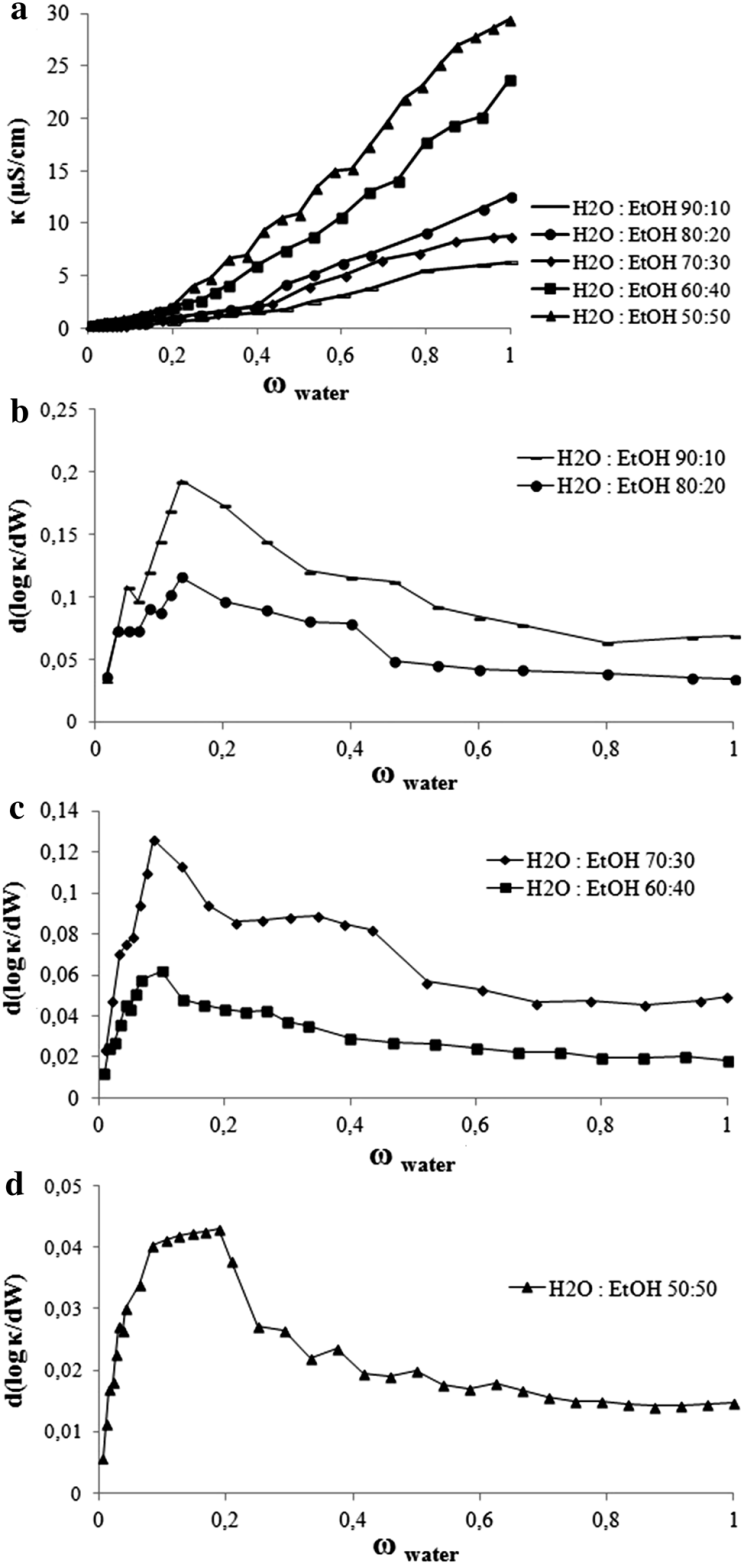
**a** The electric conductivity κ of the microemulsions as a function of water content (*W* wt%) in the system of hexadecane/MAG/PG/water:EtOH, **b–d** plot of d(log κ)/d(*W*) as a function of the water content for the systems presented in (**a**)

The electrical conductivity of the microemulsions was performed by introducing 0.05 % NaCl solution instead of the water phase (small amount of electrolyte added to the water used in the formulation of the microemulsions has no effect on the area of the one phase microemulsion region) to the systems of hexadecane/water/ethanol/MAG/PG along the *X* dilution line. The curves of electrical conductivity versus NaCl solution content increased gradually as the water phase was increasing, as shown in Fig. 4. However, even after about 20 % solubilization of water, the system conductivity did not exceed 30 μS/cm.

Figure 4b–d shows a plot of d(log κ)/d(*W*) as a function of the water content for the systems presented in Fig. 3a, depending on the EtOH concentrations (a model for the explanation of the behavior of the slope d(log κ)/d(*W*) as a function of the water fraction was initially shown in our previous work [18]). Occurrence of the changes (the maximum) have been attributed to the occurrence of a percolation transition. Our systems presented a maximum when the water phase (calculated without ethanol) amounts were: 2.9; 2.3; 2.0; 1.7 and 3.1–6.5 % in ME with: 10; 20; 30; 40 and 50 % ethanol respectively. In such involvement of the aqueous phase, systems are changing the w/o structure into bicontinuous.

The research shows that ethanol strongly affects the microstructure transition, because, as it was shown in our previous work [18], the same systems but without ethyl alcohol did not present a maximum, because they were probably already percolated, even with 1 % of the aqueous phase. This phenomenon can be explained by the lower concentrations of water added to the system when the aqueous phase was a solution of ethanol and water (electrolyte in water). But probably there is also some other mechanism at work there. At low water phase fractions, the interfacial film is too rigid and prevents the micelles to merge. The solution consists of discrete water droplets within a continuous oil phase. With the addition of more cosolvent (EtOH from 10 to 40 %) more ethanol molecules participate in the interfacial film and reduce the interfacial rigidity and favor the formation of bicontinuous ME with a lower water content. Only in the ethanol-rich area is the formation of bicontinuous structures possible, which can be explained by the less rigid interfacial film due to the intercalation of ethanol. This data is consistent with previous publications on the effects of ethanol on the percolative behavior [16, 17]. However, this behavior works only to a certain extent.

When the system contains 50 % EtOH solution, a percolation transition occurs with a larger amount of water. Below the percolation threshold, the conductivity of the microemulsion can be explained by the migration of charged aqueous droplets in the electric field. The water added to the system was first used to hydrate the polar headgroups of monoacylglycerol and propylene glycol (OH) with hydrogen bond. The ethanol molecules are also solvated by water molecules. The water was bound with OH and there was little free water in the droplet cores. Although the droplets interacted with each other, there was no free water as a medium for Na^+^ or Cl^−^ exchange. The conductivity of the microemulsions was small and did not vary much with water content increases before the hydration of OH was complete. Conversely, after the hydration had been completed, more and more free water in the droplet cores acted as a medium for Na^+^ or Cl^−^ exchange. At higher water content, the collision of water drops increases in the continuous oil phase and this forms infinite clusters of droplets. Then the conductivity displays an increase. So the percolation threshold occurs not only by reducing the interfacial film rigidity with the addition of EtOH, but also through the hydration saturation [28]. It has been indicated that continuous aqueous phase formed with droplets cluster growth due to the attractive interaction between the spherical microdroplets of the water phase and upon further dilution, the system turns into a bicontinuous microemulsion.

**Fig. 5:**
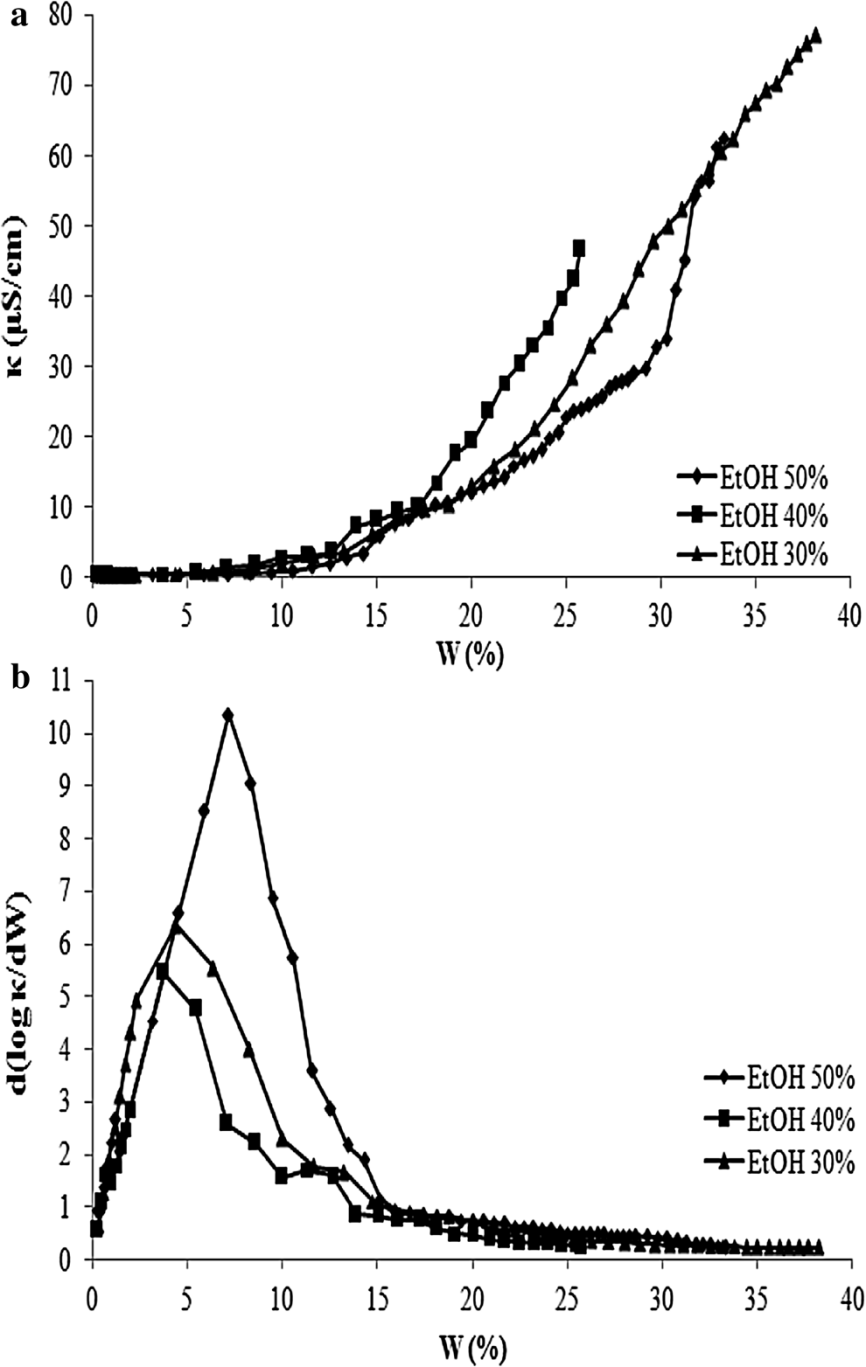
**a** The electric conductivity κ of the microemulsions as a function of water content (*W* wt%) in the system of hexadecane/G/MAG/water:EtOH; **b** plot of d(log κ)/d(*W*) as a function of the water content for the systems presented in (**a**)

As we can see from the curves obtained for the system with glycerol (Fig. 5b), percolation transition occurred when the water phase amounts were: 4.4; 3.8 and 7.1 % in ME with: 30; 40 and 50 % of ethanol, respectively. These values are higher than for the systems with propylene glycol probably due to the greater amount of hydration of the OH groups in the cosurfactant molecules (glycerol) and more surfactant molecules (MAG). As mentioned above, glycerol is known as a good cosolvent but not as a surface activity agent, and it is not incorporated into the surfactant film. With addition of short alcohol, the attractive interactions are high enough to promote (above a water content corresponding to a percolation threshold) the formation of w/o micelles whose flexible surface active agent shells facilitate electrical charge exchanges and transfers throughout the medium. In the systems with 10 and 20 % ethanol there were no changes in the structure, because the conductivity of these systems was close to zero and ME contained amounts of the aqueous phase that were too small (water with ethanol).

### DSC Measurements

It was also interesting to explore the effect of water-soluble polyol such as propylene glycol and glycerol on the thermal behavior at sub-zero temperatures. DSC is widely used for studying low-temperature behavior of surfactant-based multicomponent microemulsion systems [30, 31]. The classifications of three types of water when describing the state of water in microemulsions, as suggested by Senatra et al. [32], will be used in the present work. The differentiation is based on difference in melting (freezing) points of: (a) free water which melts at ~0 °C; (b) interfacial water, defined as water confined within the interface of the dispersed system (melts at about −10 °C) [29, 31]; (c) bound water, which is associated to hydrophilic groups (melts at <−10 °C).

**Fig. 6:**
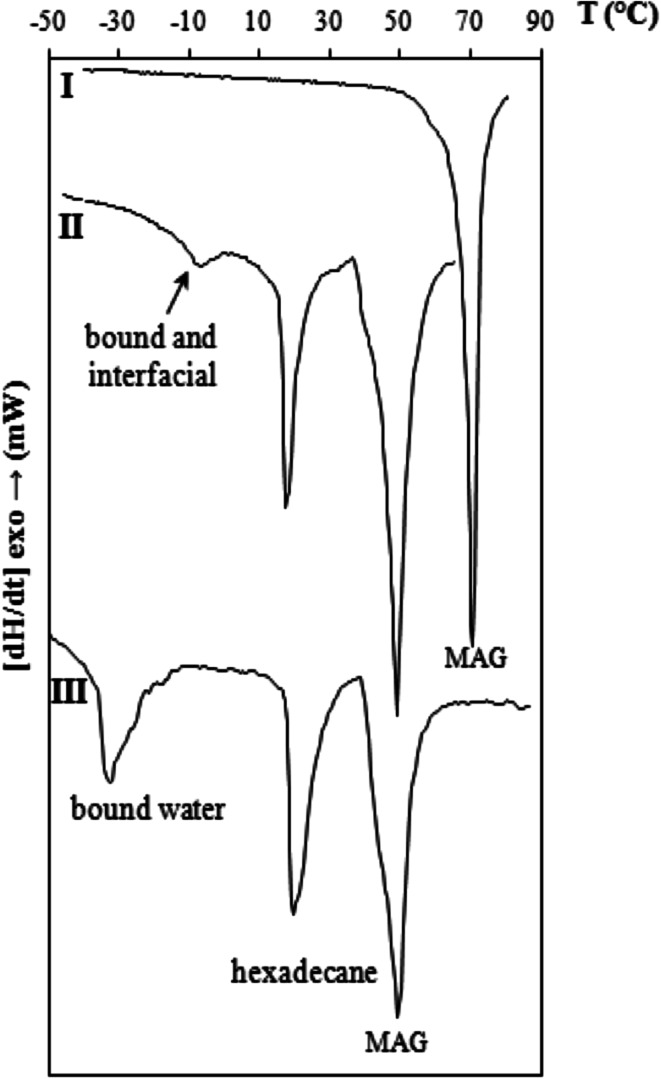
Thermogram for: *I* pure MAG; *II* system of hexadecane/MAG/PG/water; *III* system of hexadecane/MAG/PG/water:EtOH 50:50 %. All microemulsion systems consist of the maximum amount of water

Figure 6 demonstrates the thermal behavior of the pure MAG surfactant and the system of hexadecane/MAG/PG/water, hexadecane/MAG/PG/water:EtOH 50:50 %, in which the concentration of water phase was maximum. Pure MAG (peak I) melting at 70.6 °C. When we use MAG in the microemulsion system (peak II), the endothermic peak of this surfactant is shifted to a lower temperature 48.9 °C (lower melting point). The hexadecane endothermic melting peak appears at 17.5 °C. We suggest that the peak at the range −30 to −6.7 °C may be due to interfacial (bound water weakly interacting with the surfactant) and bound water.

In the study of ME with ethanol, no endothermic melting peaks of water over a temperature range were found, except the system with 50 % of EtOH (Fig. 6 III). In order to understand the effect of the solubilized aqueous phase in the microemulsion, Yaghmur and co-workers [33] prepared a binary mixture of water–PG with increasing PG content and its thermal behavior was determined. They have found that water strongly binds to PG and the melting event of the binary system, containing 50 mass% PG, is lowered to –31.2 ± 1 °C and after 80 mass% PG all the water in the system was non-freezable.

From the calculation of the composition of studied microemulsion, as can be seen in the Table 3, it follows that almost every system contained an excess of PG in relation to the water, therefore there was no melting point of water phase. An exception is the system with 50 % EtOH solution in which endothermic melting peak appears at around −35 °C (bound water) on the thermograme.

**Table 3:** Composition of the systems: hexadecane/MAG:PG/H_2_O:EtOH with maximum solubilization of aqueous phase

Solution EtOH (%)	Weight proportion (%) GP:H_2_O:EtOH
10	16:9:1
20	8:4:1
30	3.5:2.3:1
40	2:1.5:1
50	1:2:2

**Fig. 7:**
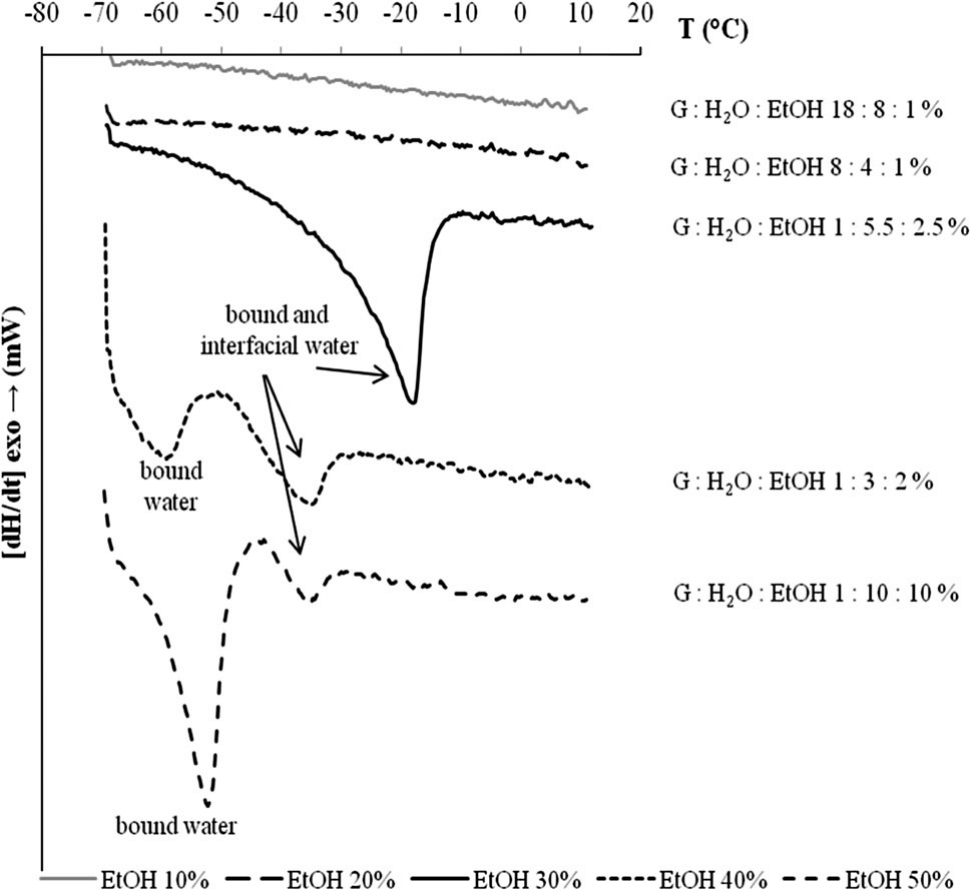
Thermograms for the system of hexadecane:G/MAG/water:EtOH. All microemulsion systems have the maximum amount of water (Table 1)

In the present study on ME with glycerol as cosurfactant (Fig. 7), no endothermic peak was found at two ethanol contents, indicating that all the water in those systems was non-freezable. When the amount of glycerol was decreased (systems with 30, 40, 50 % EtOH), the melting points of water phase were observed. We suggest that the peak at the range −40 to −10 °C may be due to interfacial and bound water. The bound endothermic melting peak of water appears also in the range −70 to −50 °C. These results differ from the range of Senatra *et al*. However, the surfactant, as expected, binds water to its hydroxyl groups. The ethanol also binds to water and so does propylene glycol or glycerol. In both cases, the hydroxyl groups reduce the melting point of the aqueous phase to lower temperatures. Water-soluble polyols such as propylene glycol (1,2-propanediol) and glycerol are cryoprotectants protecting biological systems from massive ice crystallization [34], since they lower the freezing point of water [34–37].

## Conclusion

Microemulsions based on monoacylglycerols, safe nonionic surfactants in the presence of propylene glycol, glycerol and different concentration of ethanol, were investigated concerning their ability to solubilize large water phase and were formulated and characterized for the first time. Our results show that the addition of a food grade surface active agent to the oil phase and of a polyols (such as PG and G) with a short-chain alcohol (ethanol) in the aqueous phase increased the microemulsion area significantly. Optimal composition of the polar and apolar phases led to the formation of a large isotropic area with a high content of solubilized water with an approximately low surfactant amount. These microemulsions form bicontinuous microstructures. The results of electrical conductivity showed that the electrical percolation threshold is dependent on reducing the interfacial film rigidity with the addition of EtOH and the hydration of polar headgroups in the whole system. In addition, the surfactant/alcohol/polyol can strongly bind water in the inner phase so that it freezes below −10 °C and acts in part as ‘bound’ water and in part as ‘non-freezable’ water. The results indicate that a ME of oil/MAG/PG or G/water:EtOH, may be a promising microdispersion for the protection of biological or other active materials in cosmetic, detergent, and food products, and in my further research, systems with triglycerides (vegetable oils) will be obtained.
